# 262 Looking Behind to Move Forward: Evaluating Retrospective Chart Review in Contact Precaution Discontinuation Policies

**DOI:** 10.1017/ash.2026.10628

**Published:** 2026-06-23

**Authors:** Loreen Herwaldt, Nathan Timbs

**Affiliations:** 1 University of Iowa Carver College of Medicine

## Abstract

**Background:** We used human factors engineering (HFE) methods to define processes of care in LTCF and assess healthcare professionals’ (HCP) hand hygiene (HH). **Methods:** We created an HFE observation template on which to record the types of items and patient sites HCP touched during care sequences (CS) and the order in which they touched them. We filled out the template as we shadowed HCP at 3 LTCF during 198 CS and entered data into Excel. We did descriptive analyses of the CS and HH compliance for the 3 facilities together and separately. We used chi-square, Fisher’s exact, Kruskal-Wallis, and Wilcoxon tests as appropriate. We used first-order Markov transition probabilities from the step-by-step CS to estimate the probability of HH before entry and exit and before and after each touch type. **Results:** We observed 63 CS at both LTCF1 and LTCF3 and 72 at LTCF2. 83 (41.9%) CS were short (1-7 touches), 51 (25.8%) were medium (8-13), and 64 (32.3%) were long (14-103). HH on exit decreased significantly from 31.2% and 39.2% after long and medium CS, respectively, to 19.3% for short CS. HH opportunities during CS comprised 82.8% of all opportunities. HH compliance was 27.8% on room entry, 28.3% on room exit, and 1.7% during CS. Overall HH compliance was low at each LTCF but it was significantly lower at LTCF3 than at LTCF1. HH on entry and exit at LTCF1 (38.1%; 38.5%) was significantly higher than at LTCF3 (20.6%; 20.6%) and insignificantly higher than at LTCF2 (25%; 27.8%). Touches of the environment, supplies/equipment, and clean patient sites were the most common touches, accounting for 55.5-70.9% of touches. Dirty/contaminated touches comprised only 4.2%- 5.8%. We observed only 2 clean/aseptic touches at LTCF3. The highest transition probabilities from specific touches to HH were from the environment (0.460) and from supplies/equipment (0.264) touches and the lowest were from dirty/contaminated and from HCP’s sites (both 0.011). The highest transmission probabilities for touches immediately after HH were for dirty/contaminated sites (0.105) and for eating-related items (0.105) and the lowest were for clean/aseptic touches (0.000). **Conclusions:** Most CS are short and involve touches of the environment, supplies/ equipment, and clean patient sites. Low HH compliance on exit and entry increase the risk of pathogen transmission among residents. Although dirty/contaminated touches and clean/aseptic touches were rare, HH during CS was also rare and the transition probabilities indicate that HH compliance is low at critical care transitions.

